# The impact of rural residence and HIV infection on poor tuberculosis treatment outcomes in a large urban hospital: a retrospective cohort analysis

**DOI:** 10.1186/s12939-017-0714-8

**Published:** 2018-01-08

**Authors:** Aishatu Lawal Adamu, Muktar H. Aliyu, Najiba Aliyu Galadanci, Baba Maiyaki Musa, Umar Muhammad Lawan, Usman Bashir, Ibrahim Abubakar

**Affiliations:** 1 Department of Community Medicine, College of Health Sciences, Bayero University Kano, Kano, Nigeria; 2 Department of Community Medicine, Aminu Kano Teaching Hospital, Kano, Nigeria; 3 Department of Health Policy, Vanderbilt University School of Medicine, Nashville, TN USA; 4 Vanderbilt Institute of Global Health, Nashville, TN USA; 5 Department of Haematology, Aminu Kano Teaching Hospital, Kano, Nigeria; 6 Department of Medicine, College of Health Sciences, Bayero University Kano, Kano, Nigeria; 7 Institute for Global Health, University College London, London, UK

**Keywords:** Tuberculosis, Nigeria, Treatment outcome, Poor treatment outcome, Rural residence, HIV

## Abstract

**Background:**

Successful tuberculosis (TB) treatment is essential to effective TB control. TB-HIV coinfection, social determinants and access to services influenced by rural residence can affect treatment outcome. We examined the separate and joint effects of rural residence and HIV infection on poor treatment outcome among patients enrolled in a large TB treatment centre in Kano, Nigeria.

**Methods:**

We retrospectively analysed a cohort of patients with TB enrolled in a large urban TB clinic in northern Nigeria, from January 2010 to December 2014. Poor treatment outcome was defined as death, default or treatment failure. We used Poisson regression to model rates and determine the relative risks (and 95% confidence intervals, CI) of poor treatment outcomes.

**Results:**

Among 1381 patients included in the analysis, 28.4% were rural residents; 39.8% were HIV-positive; and 46.1% had a poor treatment outcome. Approximately 65 and 38% of rural and urban residents, respectively, had a poor treatment outcome. Rural residents had 2.74 times (95% CI: 2.27–3.29) the risk of having a poor treatment outcome compared to urban residents. HIV-positive patients had 1.4 times (95% CI: 1.16–1.69) the risk of poor treatment outcome compared to HIV-negative patients. The proportion of poor treatment outcome attributable to rural residence (population attributable fraction, PAF) was 25.6%. The PAF for HIV infection was 11.9%. The effect of rural residence on poor treatment outcome among HIV-negative patients (aRR:4.07; 95%CI:3.15–5.25) was more than twice that among HIV-positive patients (aRR:1.99; 95%CI:1.49–2.64).

**Conclusion:**

Rural residents attending a large Nigerian TB clinic are at increased risk of having poor treatment outcomes, and this risk is amplified among those that are HIV-negative. Our findings indicate that rural coverage of HIV services may be better than TB services. These findings highlight the importance of expanding coverage of TB services to ensure prompt diagnosis and commencement of treatment, especially among rural-dwellers in resource-limited settings.

## Background

Effective tuberculosis (TB) control is largely dependent on successful treatment. Between 2000 and 2014, 43 million deaths were prevented through effective TB treatment and global TB deaths have declined by almost half from 1990. [1] Despite this progress, each year over 1 million deaths from TB occur and about 3.6 million people with TB are still missed by health systems annually and therefore fail to receive appropriate care. [2] The importance of identifying sub-groups with high risk of TB and its consequences has been emphasised. [3, 4] The End TB Strategy recognises the need to address underlying social determinants (poverty, food insecurity, poor living and working conditions) and consequences (catastrophic economic costs, stigmatisation, social isolation, loss of job and divorce) of TB to effectively control the disease. [5–7] HIV infection, one of the major drivers of TB burden in sub-Saharan (SSA) also complicates diagnosis and treatment of TB, and is associated with poorer treatment outcomes, such as treatment failure and death. Early diagnosis and prompt initiation of treatment with anti-tuberculous medications reduce infectiousness, transmission, deaths and other poor outcomes, including treatment failure and drug resistance.

Nigeria is a high burden country with regards absolute number of TB cases, TB-HIV co-infection and multi-drug resistance. [8] An estimated 586,000 new TB cases (322/100,000 population) and 240,000 TB-related deaths occur in Nigeria yearly. [9] TB control in Nigeria is managed by the TB and Leprosy Control Programmes operating at the three levels of healthcare. [10] TB in Nigeria is largely externally funded through the Global Fund to fight AIDS, TB and Malaria (GFATM), and the United States Agency for International Development (USAID) through Challenge TB; and to a lesser extent domestically, with persistent funding gaps especially at lower government levels. [11–13] TB treatment and microscopy services are provided free across treatment centres and microscopy sites in the country, [14] with lower coverage in the northern states. [11] TB services are also disproportionately distributed in favour of urban areas, [14] potentially leaving a larger proportion of people from rural areas with poor access to TB care. Geographic barriers related to rural living influence access to TB care and clinical outcomes. [3] Additionally, individual risk factors of rural residents such as low educational attainment and income can influence care-seeking behaviours, resulting in treatment delays and poor treatment adherence. [15] Poor access to health care could also lead patients to seek less credible alternative care. [16] Though urban residence is a recognised risk factor for TB, especially in rapidly urbanising communities due to poor living conditions, [3] the gap in TB services coverage between the northern and southern part of the country, as well as between the rural and urban areas may have worsened inequalities to treatment access which can affect the treatment outcome. In addition to providing specialist services such as diagnosis of extra-pulmonary and smear-negative TB, several tertiary-level facilities in the country (which are largely urban-based) also provide primary care services, such as diagnosis and treatment of pulmonary TB. This imbalance may also further worsen rural-urban inequalities in TB care. Establishing the effect of rural residence in the context of the country with the 4th largest global burden of TB and gross inequalities in TB services, while adjusting for confounding factors, which previous studies have not always done, provides important policy-relevant information.

Due to high TB-burden and disproportionately distributed TB services in Nigeria, we anticipate that a substantial number of people in rural areas would need to access care in large treatment centres within cities. The aim of this analysis was to describe treatment outcomes and examine the impact of rural residence and HIV infection on poor treatment outcomes among TB patients attending a large urban treatment centre. Our hypothesis is that rural residents who travel to access care in urban centres have a higher risk of poor TB treatment outcome, and this risk is modified by their HIV status. [3]

## Methods

### Study population

Data from patients age ≥ 15 years and enrolled in Aminu Kano Teaching Hospital (AKTH) TB clinic for TB treatment between 2010 and 2014 were included in this analysis. A total of 43 (2.9%) of 1424 patients that were transferred to another treatment centre were excluded. Patients diagnosed with TB received treatment based on the existing national TB treatment guidelines. [17, 18] Recommended treatment duration was either 8 months (up to 2012) or 6 months (from 2013), except for some cases of extrapulmonary TB - spine and central nervous system (CNS), where treatment is longer. Treatment comprised four drugs – Rifampicin (R), Isoniazid (H), Pyrazinamide (Z) and Ethambutol (E) in the 2-month intensive phase; and two drugs in the continuation phase- either Ethambutol and Isoniazid (up to 2012) or Rifampicin and Isoniazid (from 2013 onwards). Before drug susceptibility testing became available in the hospital (2014 onwards), treatment regimen for re-treatment patients comprised 3-month intensive phase with RHZE with Streptomycin added in the first 2 months; and 5-month continuation phase with RHE. Patients enrolled in the clinic included presumed and confirmed TB cases referred from the same hospital or other hospitals within and outside Kano. TB/HIV services are co-located and patients are routinely counselled and screened for HIV, if their HIV status is unknown.

### Study design

This study was a retrospective cohort design that included patients who commenced TB treatment from January 2010 to December 2014. Cohort entry was defined as the date of treatment initiation. Cohort exit was defined as the first to occur from: treatment completion, death prior to treatment completion, treatment failure and loss to follow-up. Follow-up time was ordered by time since treatment initiation, and tracked as person-months (pm).

Data on independent variables and treatment outcome were collected from clinic-based records. Information available from the records included: date of treatment onset, age, sex, place of residence, mode of diagnosis, disease site, referral source, HIV status at treatment onset, history of previous TB treatment and treatment outcome. Medication intake was observed in the clinic once a week during the intensive phase, and once a month during the continuation phase. Daily supervision of medication intake was performed by an assigned family member. Data on independent variables were not updated during follow-up. Site of disease was grouped as pulmonary (disease affecting lungs only), extra-pulmonary (disease affecting organs other than the lung) and both (disease affecting the lungs and any other organ). The rationale for grouping the disease site into three and not as done in the National guidelines (where patients with both pulmonary and extrapulmonary TB are classified as pulmonary TB), was to allow us to account for disease severity, as patients with TB involving 2 or more organs tend to have more severe disease. Mode of diagnosis was grouped as bacteriologically-diagnosed (sputum-smear or culture confirmed) or clinically diagnosed (smear-negative and physician-diagnosed using clinical features with radiological and/or other laboratory evidence and a decision to treat with at least 6 months anti-TB therapy).

The outcome variable was poor treatment outcome, which was a composite measure comprising death from any cause including deaths from TB among HIV-positive persons; treatment failure from lack of conversion or reversion; and loss to follow-up (LTF) from interruption of treatment for two consecutive months or more. [19–22] Outcome was ascertained for 97% of the patients.

The primary exposure variables were: place of residence, classified as urban (for patients residing in Kano city) and rural (for patients residing outside Kano city); and HIV status as at time of treatment onset, classified as HIV-positive, HIV-negative and unknown HIV status.

### Statistical analysis

We summarised categorical variables using frequencies and proportions. We described the distribution of explanatory variables in the whole study population and stratified by treatment outcome status. We also described the distribution of all potential confounders across strata of primary exposures (residence and HIV). Univariable analysis was performed by cross-tabulating independent variables with treatment outcome, and association assessed one at a time using chi-squared test.

We examined association between poor treatment outcome and exposure variables (residence and HIV) using Poisson regression models. Age, sex and year of treatment onset were denoted as forced variables and a priori included in the model. Variables that were associated with the outcome after adjusting for age, sex and calendar year at *p* < 0.2 were included in the multivariable analysis. A multivariable model was built in a forward stepwise manner, one variable at a time. Potential confounders were added sequentially and were retained in the model if they notably changed the effect estimate (relative risk, RR) by at least 10%.

We estimated the population attributable fraction (PAF) of rural residence and HIV status from the final multivariate model by using the standard formula:

PAF = p’(θ − 1)/ θ.

where p’ denotes the respective proportions of persons with poor outcome who were rural residents or HIV-positive and θ represents the relative risks (RR) from the multivariable model.

We also examined for interaction between HIV status and rural residence and between sex and rural residence by fitting an interaction term in the multivariable models. We used a likelihood ratio test to compare the model with and without interaction and presented stratum-specific estimates.

All analyses were performed using Stata 14 (Stata Corp, College Station, TX, USA). This study was approved by the Institutional Review Board of Aminu Kano Teaching Hospital, Nigeria.

## Results

We analysed data on 1381 TB cases enrolled between January 2010 and December 2014. More than two-thirds of the participants were between 15 and 44 years at the time of treatment commencement. More than half (56.8%) were males. Pulmonary disease was the most common form of TB in participants (65.7%). Common extra-pulmonary sites comprised the abdomen, spine, and the CNS. Other less common sites included the skin, larynx and adrenals. At the time of treatment commencement, 392 (28.4%) patients were rural residents and 550 (39.8%) were co-infected with HIV, of which only 90 (16.4%) were on anti-retroviral treatment (ART). The distribution of age, HIV status, sex, and TB site of the 43 excluded patients were similar to those included in this analysis. However, the patients excluded were more likely to be rural residents (41.9%), and less likely to have a history of prior TB (4.7%).

Nearly half of the patients (46.1%) had a poor treatment outcome after a total follow-up time of 6377.2 person-months (pm), giving a rate of 9.9/100 pm (95% CI: 9.2–10.8). Of the 636 patients with poor outcome, 57.5% were lost to follow-up, 37.3% died during treatment, and 5.2% failed treatment.

Crude analysis showed that nearly two-thirds (65.3%) of the rural residents had a poor treatment outcome of treatment, compared to 38.4% of urban residents. When poor outcome was further broken down to specific treatment outcome, among urban residents, 16.7% were lost to follow-up, 3.0% failed treatment and 8.9% died during treatment. While among rural residents, 13.1% were lost to follow-up, 2.0% failed treatment and 46.0% died. The distribution of explanatory variables across treatment outcomes is shown in Table 1.

**Table 1:** Crude association between poor treatment outcome and potential confounders, TB clinic, Aminu Kano Teaching Hospital, Kano, Nigeria

Variable	Total (%)	Poor outcome		*P* value (χ^2^)
		Yes (%)	No (%)	
Age group (years)				
15–24	252	91 (36.9)	159 (63.1)	
25–34	382	169 (44.2)	213 (55.8)	
35–44	323	139 (43.0)	184 (57.0)	
45–54	181	105 (58.0)	76 (42.0)	
55–64	82	45 (54.9)	37 (45.1)	
> 65	70	44 (62.9)	26 (37.1)	<0.001
Sex				
Female	785	259 (43.5)	337 (56.5)	
Male	596	377 (48.0)	408 (52.0)	0.005
Residence				
Urban	989	380 (38.4)	609 (61.6)	
Rural	392	256 (65.3)	136 (34.7)	<0.001
Referral facility				
DOTS-linked facility	611	245 (40.1)	366 (59.9)	
Non DOTS-linked facility	710	371 (52.3)	339 (47.7)	<0.001
TB confirmation				
Bacteriological	461	126 (27.3)	335 (72.7)	
Clinical	920	510 (55.4)	410 (44.6)	<0.001
TB site				
Pulmonary	912	370 (40.6)	542 (59.4)	
Extra-pulmonary	220	95 (43.2)	125 (56.8)	
Both	213	140 (65.7)	73 (34.3)	<0.001
HIV/ART status				
HIV-	662	292 (44.1)	370 (55.9)	
HIV+	550	264 (48.0)	286 (52.0)	
Unknown HIV status	169	80 (47.3)	89 (52.7)	0.008
Previous TB treatment				
No	1041	411 (39.5)	630 (33.8)	
Yes	340	225 (66.2)	115 (33.8)	<0.001

Univariable associations between residence and other potential confounders (Table 2) showed that the majority (81.4%) of rural residents had a clinical diagnosis, compared to 60.8% of urban residents. Nearly half (42.9%) of rural residents had a history of previous TB treatment compared to 17.4% of urban residents. The distribution of rural residence was similar (*p* = 0.14) among patients who were HIV-negative (28.3%), HIV-positive (30.4%) or had unknown HIV status (22.5%). (Table 3).

**Table 2:** Association between participant’s residence and covariates, TB clinic, Aminu Kano Teaching Hospital, Kano, Nigeria

Variable	Urban (%)	Rural (%)	*P* value (χ^2^)
Age group (years)			
15–24	196 (20.9)	56 (15.9)	
25–34	291 (31.1)	91 (25.8)	
35–44	237 (25.3)	86 (24.4)	
45–54	114 (12.2)	67 (19.0)	
55–64	56 (6.0)	26 (7.4)	
> 65	43 (4.6)	27 (7.7)	0.002
Sex			
Male	530 (53.6)	255 (65.1)	
Female	459 (46.4)	137 (34.9)	<0.001
Referral facility			
DOTS-linked facility	484 (48.6)	127 (33.5)	
Non DOTS-linked facility	458 (51.4)	252 (66.5)	<0.001
TB confirmation			
Bacteriological	388 (39.2)	73 (18.6)	
Clinical	601 (60.8)	319 (81.4)	<0.001
TB site			
Pulmonary	707 (73.4)	205 (53.7)	
Extra-pulmonary	155 (16.1)	65 (17.0)	
Both	101 (10.5)	112 (29.3)	<0.001
HIV status			
HIV-	475 (48.0)	187 (47.7)	
HIV+	383 (38.7)	167 (42.6)	
Unknown HIV status	131 (13.3)	38 (9.7)	0.02
Previous TB treatment			
No	817 (82.6)	224 (57.1)	
Yes	172 (17.4)	168 (42.9)	<0.001
Treatment outcome			
Cured	281 (32.9)	47 (13.4)	
Treatment completed	328 (38.4)	89 (25.4)	
Lost to follow-up	143 (16.7)	46 (13.1)	
Died	76 (8.9)	161 (46.0)	
Treatment failed	26 (3.0)	7 (2.0)	<0.001

**Table 3:** Association between participant’s HIV status on enrolment and covariates, TB clinic, Aminu Kano Teaching Hospital, Kano, Nigeria

Variable	HIV-negative (%)	HIV-positive (%)	HIV status unknown (%)	*P* value (χ^2^)
Age group (years)				
15–24	166 (26.8)	58 (11.3)	28 (18.1)	
25–34	181 (29.2)	156 (30.3)	25 (29.0)	
35–44	112 (18.1)	181 (35.2)	30 (19.4)	
45–54	67 (10.8)	86 (16.7)	28 (18.1)	
55–64	46 (7.4)	23 (4.5)	13 (8.4)	
> 65	48 (7.7)	11 (2.1)	11 (7.1)	<0.001
Sex				
Male	395 (59.7)	296 (53.8)	94 (55.6)	
Female	267 (40.3)	254 (46.2)	75 (44.4)	0.12
Referral facility				
DOTS-linked facility	423 (63.9)	212 (42.8)	75 (45.7)	
Non DOTS-linked facility	239 (36.1)	283 (57.2)	89 (54.3)	<0.001
TB confirmation				
Bacteriological	239 (36.1)	172 (31.3)	50 (29.6)	
Clinical	423 (63.9)	378 (68.7)	119 (70.4)	0.11
TB site				
Pulmonary	406 (62.5)	386 (72.3)	120 (74.5)	
Extra-pulmonary	120 (18.5)	71 (13.3)	29 (18.0)	
Both	124 (19.1)	77 (14.4)	12 (7.5)	<0.001
Residence				
Urban	475 (71.8)	383 (69.6)	131 (77.5)	
Rural	187 (28.3)	167 (30.4)	38 (22.5)	0.14
Previous TB treatment				
No	503 (76.0)	395 (71.8)	143 (84.6)	
Yes	159 (24.0)	155 (28.2)	26 (15.4)	0.003
Treatment outcome				
Cured	178 (30.7)	116 (23.9)	34 (24.3)	
Treatment completed	192 (33.2)	170 (35.1)	5 (39.3)	
Lost to follow-up	81 (14.0)	75 (15.5)	33 (23.6)	
Died	111 (19.2)	111 (22.9)	15 (10.7)	
Treatment failed	17 (2.9)	13 (2.7)	3 (2.1)	0.006

The rate of poor outcome was higher among rural residents (21.1/100 pm;95% CI:18.7–23.8) than in urban residents (7.4/100 pm;95% CI: 6.6–8.1). The rate of poor outcome was also higher among HIV-positive patients (11.4/100 pm;95% CI: 10.1–12.8) compared to HIV-negative patients (9.0/100 pm;95% CI: 8.0–10.1). (Table 4).

**Table 4:** Univariable and Multivariable analyses of effect of rural residence and poor treatment outcome using Poisson regression showing crude and adjusted relative risks (RR) with 95% CIs, TB clinic, Aminu Kano Teaching Hospital, Kano, Nigeria

	No with poor outcome	Person-months of follow-up	Rate (95% CI)	Crude RR (95% CI)	Minimally-adjusted RR (95% CI)^a^	Fully adjusted RR (95% CI)^b^
Residence						
Urban	380	5163.4	7.4 (6.6–8.1)	1	1	1
Rural	256	1213.8	21.1 (18.7–23.8)	3.21 (2.71–3.81)	3.43 (2.89–4.07)	2.74 (2.27–3.29)
HIV status						
HIV-negative	292	3252.2	9.0 (8.0–10.1)	1	1	1
HIV-positive	264	2319.3	11.4 (10.1–12.8)	1.27 (1.07–1.50)	1.71 (1.43–2.06)	1.40 (1.16–1.69)
HIV status unknown	80	805.7	9.9 (8.0–12.4)	1.11 (0.86–1.42)	0.98 (0.75–1.29)	0.85 (0.64–1.12)

^a^adjusted for age, sex and calendar year

^b^adjusted for age sex, calendar year, referral facility, TB confirmation mode, TB site, HIV status, and previous TB treatment status

In multivariable analysis, after adjusting for age, sex, calendar year, mode of diagnosis, TB site, HIV status, prior TB history, and referral source, rural residents had 2.74 times (95% CI:2.27–3.29) the risk of a poor treatment outcome compared to urban residents; while HIV-positive patients had 1.4 times (95% CI:1.16–1.69) the risk compared to HIV negative patients (Table 4). Using the relative risks from the fully-adjusted model, the estimated PAFs were 25.6 and 11.9% for rural residence and HIV infection respectively.

**Fig. 1:**
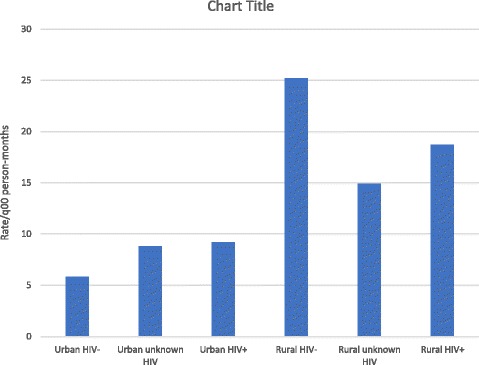
Rates of poor treatment outcome according to HIV status and residence

**Fig. 2:**
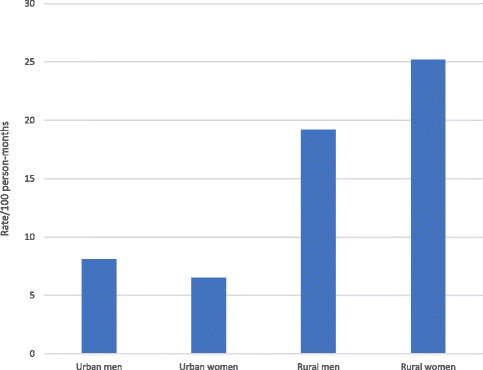
Rates of poor outcome according to sex and residence

We examined the joint effects of HIV infection and sex with place of residence. Within each strata of HIV status and sex, rates of poor outcome were higher in rural residents. However, rates were highest among HIV-negative rural residents and rural women respectively. (Figs. 1 and 2) We found strong evidence of interaction between rural residence and HIV status (*p* < 0.001), and between rural residence and sex. Across strata of HIV status, effect of rural residence was higher among HIV-negative patients and women. (Table 5) Adjusted stratum-specific RRs showed that among HIV negative patients, rural residents had more than 4 times (RR:4.07;95% CI:3.15–5.25) the risk of poor treatment outcome, while among HIV-positive patients, rural residents had about twice the risk (RR:1.99;95% CI:1.49–2.64).

**Table 5:** Effect of rural residence on poor treatment outcome stratified by HIV-status and sex showing stratum-specific relative risks (RR) with 95%CIs, TB clinic, Aminu Kano Teaching Hospital, Kano, Nigeria

Variable	Residence	No with poor outcome/person-months	Stratum-specific crude RR for poor treatment outcome (95% CI)	Stratum-specific adjusted RR for poor treatment outcome (95% CI)^a^	*P*-value for interaction (LRT)^b^
HIV status					
Negative	Urban	159/2725.2	1	1	
	Rural	133/526.9	4.32 (3.44–5.45)	4.07 (3.15–5.25)	
Positive	Urban	163/1780.1	1	1	
	Rural	101/539.3	2.05 (1.60–2.62)	1.99 (1.49–2.64)	
Unknown	Urban	58/658.1	1	1	
	Rural	22/147.6	1.69 (1.04–2.76)	1.55 (0.88–2.73)	<0.001
Sex					
Male	Urban	218/2678.7	1	1	
	Rural	159/829.1	2.36 (1.92–2.89)	2.16 (1.71–2.72)	
Female	Urban	162/2484.7	1	1	
	Rural	97/384.7	3.87 (3.01–4.97)	4.08 (3.06–5.44)	0.001

^a^adjusted for age, sex, calendar year, referral facility, TB confirmation mode, TB site, HIV status, and previous TB treatment status

^b^Likelihood ratio test

## Discussion

In this study, we found high levels of poor treatment outcome among a cohort of patients attending a TB clinic in urban Kano, Nigeria. We show that rural residents have more than twice the risk of having a poor treatment outcome when compared to urban residents, and this relationship is modified by HIV status and sex. HIV-positive patients also have a higher risk of poor treatment outcome compared to HIV-negative patients. Our findings show that rural residents who are HIV-negative are substantially more at risk of poor treatment outcomes than their HIV-positive counterparts. Rural women also have a higher risk of poor treatment outcomes compared to rural men.

We found rural residence to be an independent risk factor for poor TB treatment outcome. Though TB prevalence has been shown to be higher in urban settings, [23] several factors could explain poor treatment outcome among rural residents, including: low individual and household income, lower level of education, limited access to health-care, and comorbid illnesses. In addition to poor coverage of TB services in rural areas, rural residents may have to incur extra out-of-pocket cost travelling to urban areas to access care. Though drugs and microscopy services are provided free by the National TB programme, hidden costs such as lack of transportation, bad/inaccessible roads, and need for supportive investigations may contribute to poor treatment-seeking behaviour, or cause missed clinic visits. [3, 24] In poor households, especially in rural areas, choices have to be made on how to spend scarce household resources, especially in the presence of competing priorities. [25] Health seeking may therefore be delayed for household members and treatment adherence may be compromised. Lost productivity on days patients have to travel to the city to access care may additionally affect household income. Attitudes including fear of stigma can affect health seeking behaviours and adherence to treatment. [26, 27] Women also have poorer socio-economic status, treatment-seeking behaviours, and healthcare access, and hence higher risk of poor treatment outcomes. [3] Large patient turnout and long waiting periods in large hospitals such as the study site may deter patients from seeking care, and has been shown to be associated with treatment default. [28] Such a situation may lead to patients seeking care from unskilled sources and further delay diagnosis and treatment. A TB prevalence survey in Nigeria showed that among suspected TB cases, rural residents were more likely to consult traditional healers while urban residents were more likely to consult pharmacy shops, commonly termed ‘chemists’. [23] The use of traditional herbal medications is a recognised risk factor for kidney and liver disease, [29, 30] Traditional herbal medications may also interfere with anti-TB drug metabolism with potential for reducing efficacy. [31] Moreover, traditional healers are more accessible to patients, particularly those residing in rural areas. This preference for non-orthodox health care and relatively easier access to traditional health providers may increase the likelihood of unfavourable treatment outcomes. [28] High levels of poor treatment outcome observed in this cohort could be attributed to the high prevalence of TB/HIV co-infection, poor treatment adherence, delays in diagnosis and treatment, and misdiagnosis. HIV infected persons are more likely to acquire active TB infection and progress to active disease, and those co-infected with TB are more likely to have poor treatment outcomes, [32–34] though ART use may attenuate this risk. [35, 36] Co-infection is associated with disease progression. Adverse drug interactions, coinciding toxicities, and high pill burden in co-infected patients may affect adherence or result in treatment failure. [37] Associated co-morbidities that may increase the risk of poor outcomes are also more frequent among co-infected patients. [3, 36, 38]

When we stratified our data by HIV status, the effect of rural residence among HIV-negative persons was more than twice that among HIV-positive persons. We anticipated their combined effects to be greater than a multiplicative relationship, such that patients who are both rural residents and HIV-positive would have higher risks of poor outcome, since both HIV infection and rural residence are independent risk factors for poor treatment outcome. Surprisingly, HIV-negative rural residents had highest risk of poor treatment outcome. A possible explanation of our findings of negative interaction between rural residence and HIV infection is the relative success of decentralisation of HIV care, including TB-HIV services. [39, 40] This situation may have led to quicker diagnosis and treatment among rural residents who are HIV-positive compared to those who are HIV-negative, attenuating the effect of rural residence among HIV-positive persons. [41] While HIV services are provided by higher-cadre staff, the majority of TB services especially in rural areas are provided by lower-cadre health workers, with limited capacity to diagnose smear-negative and extra-pulmonary TB. This may cause delays in TB diagnosis and treatment that contribute to unfavourable outcomes. Additionally, HIV-negative patients from outside the city may face difficulties in navigating through bureaucracies of referral in a large teaching hospital. Stratifying our data by sex, we also show that effect of rural residence on poor treatment outcomes was higher among women than men. This finding could be related to underlying biological and social vulnerability, including co-morbidities such as anaemia and undernutrition, pregnancy, geographic and socio-cultural barriers to accessing care, and economic dependency. [42–44]

Our findings indicate that rural residents are more likely to have a poor treatment outcome, however, previous investigators have shown varying associations between residence and treatment outcomes. In a UK study, patients living in rural areas were less likely to complete TB treatment compared to urban residents on crude analysis, though the association was confounded by factors such as age, sex, ethnicity, recent immigration to UK and disease site. [21] Crude assessment of TB treatment outcomes in the Solomon Islands from 2000 to 2011 showed almost similar proportions of treatment success among urban and rural areas. [45] Adjusting for age, sex, TB site and year of treatment, a 10-year retrospective analysis of treatment outcomes in Ethiopia showed that patients from rural areas were more likely to have treatment success than those from urban areas. [46] In Zimbabwe, after adjusting for age, sex and HIV-status, urban residents were more likely to have an unfavourable treatment outcome. [22] Hospital-based studies tend to show that rural residence was associated with poor treatment outcomes. [47–49] However, an analysis of treatment outcomes from two treatment centres (urban public and rural private) in southern Nigeria, revealed that poor treatment outcome was associated with receiving care at an urban public health facility. The majority of the patients in the study (88%) received care from the private health care facility (rural) and may not necessarily be residing in rural areas. [50] It is possible that our data may be missing milder cases from rural areas and we may have overestimated poor treatment outcome because milder cases are more likely to have better treatment outcomes. However, since our study site is a tertiary hospital which provides specialist care, our data will capture more severe cases irrespective of place of residence and any overestimation of poor treatment outcome will also apply to urban residents.

Assuming a causal relationship and absence of residual confounding, we found that about a quarter of poor outcomes could be averted, if the increased risk associated with rural residence could be eliminated. Decentralisation of TB care to improve access to rural residents could substantially reduce poor treatment outcomes.

Our findings have important implications for TB control. Poorer treatment outcomes such as treatment failure and loss to follow-up among rural residents can increase the risks of further transmission within the community; drug resistant TB; and catastrophic economic costs and burden from treatment of disease complications on already impoverished households. More frequent poor outcomes among the younger and probably more economically and physically productive patients could directly affect household income sources and have wider impact on the basic needs of food and healthcare of other vulnerable household members, such as young children and women. TB/HIV co-infection has been traditionally recognised as a risk factor for poor TB outcomes. However, following expansion of HIV services which include HIV-TB care, HIV-infected persons may have better access to TB care, including referral and management of other co-morbidities which may improve treatment outcomes. Our results suggest that for patients who have to travel to access TB care in a large treatment centre, being HIV-negative may actually be detrimental to treatment outcomes. We believe our findings are generalizable to many tertiary-level facilities that provide TB care across the country, and therefore the implications could have a much wider impact on TB control in Nigeria.

Our results may be explained by individual socio-cultural factors that may influence health and health care-seeking among rural residents, however, we believe the contextual effects of rural residence captures effects of both compositional individual-level and group-level factors. [15, 51] Due to the retrospective nature of this study, we were limited by information available from the TB registers, and as such we could not account for other measures of disease severity including HIV stage and other comorbid conditions. We may therefore have over-estimated the effect of rural residence on treatment outcome, if rural residents had more severe disease. However, reasons for seeking care at an urban facility may also be related to unavailability of TB care in rural areas and not only referral for more severe disease. We also did not account for anti-retroviral use among HIV-positive patients, because only ART status at treatment initiation was recorded and was not updated in the patient records if ART was commenced during TB treatment. Thus, we may have over-estimated the effect of HIV infection. It is possible that the patients excluded could have a different risk of poor treatment outcome because they were more likely to be rural residents. However, other characteristics of the excluded patients (age, sex, TB site, HIV status) were similar to those included in this analysis. There may be differences in causes and risk factors for death, loss to follow-up or treatment failure that we may not have captured in our analysis, as we grouped the different sub-categories comprising poor treatment outcome as one. Risk factors for treatment failure reported from previous studies include drug resistance, severity of disease, co-morbidities, positive culture at 2 months [52, 53]; while low socio-economic status, poor adherence, poor access to health care, substance abuse, fear of stigma, lack of social support, and anti-TB side effects have been implicated in treatment default. [27, 28] However, risk factors for different poor treatment outcomes have also been shown to overlap. [34]

## Conclusion

Our findings show high rates of poor treatment outcome in this large TB treatment centre. The effect of rural residence on poor TB treatment outcome has implications on current and future interventions. There is a need to more closely monitor rural-dwelling patients accessing care in urban centres and to further decentralise TB care to rural areas including strengthening of community-based TB services in rural areas to improve access to early diagnosis and treatment. Given that HIV services have wider coverage and are better resourced than TB services, facilities providing HIV care in rural communities can expand TB services (diagnosis and treatment) to residents irrespective of their HIV status. This expansion would reduce delays in diagnosis and treatment initiation, and the socio-economic risks and treatment outcome consequences associated with travelling to access care. Ultimately, resource-constrained countries with high burden of TB and HIV as well as other diseases should aim at integrating healthcare and minimise emphasis on vertical programmes. Though tailoring appropriate interventions toward persons at risk of poor treatment outcome may have greater impact on TB control than blanket population-wide interventions, proposed interventions should in addition to individual-level approaches also consider population-based sociocultural and economic contexts. For instance, women empowerment, and girl-child education may be important in northern Nigeria in improving the health status and treatment-seeking behaviour of women and their families. Social protection initiatives which include cash transfers, microfinance credit schemes to improve income-producing activities such as farming, and skills development and capacity building will reduce household poverty and mitigate the impact of unexpected and catastrophic costs of illness on the household. Although TB care coverage is less than satisfactory in many high TB-burden countries, it is important that patients with TB that reach the health system get a favourable treatment outcome.

## Abbreviations

AKTH: Aminu Kano Teaching Hospital
ART: Antiretroviral therapy
CNS: Central nervous system
HIV: Human immunodeficiency virus
LTF: Loss to follow-up
PAF: Population attributable fraction
PM: Person-months
RR: Relative risk
TB: Tuberculosis
